# High flow variant postural orthostatic tachycardia syndrome amplifies the cardiac output response to exercise in adolescents

**DOI:** 10.14814/phy2.12122

**Published:** 2014-08-28

**Authors:** Paolo T. Pianosi, Adele H. Goodloe, David Soma, Ken O. Parker, Chad K. Brands, Philip R. Fischer

**Affiliations:** 1 Department of Pediatric and Adolescent Medicine, Mayo Clinic, Rochester, Minnesota; 2 Division of Pulmonary and Critical Care Medicine, Mayo Clinic, Rochester, Minnesota; 3 Department of Pediatrics, Vanderbilt University, Nashville, Tennessee; 4 Department of Pediatrics, All Children's Hospital, St. Petersburg, Florida and; 5 Johns Hopkins School of Medicine, Baltimore, Maryland

**Keywords:** Cardiac output, exercise, hyperkinetic circulation, orthostatic intolerance, sympathetic nervous system

## Abstract

Postural orthostatic tachycardia syndrome (POTS) is characterized by chronic fatigue and dizziness and affected individuals by definition have orthostatic intolerance and tachycardia. There is considerable overlap of symptoms in patients with POTS and chronic fatigue syndrome (CFS), prompting speculation that POTS is akin to a deconditioned state. We previously showed that adolescents with postural orthostatic tachycardia syndrome (POTS) have excessive heart rate (HR) during, and slower HR recovery after, exercise – hallmarks of deconditioning. We also noted exaggerated cardiac output during exercise which led us to hypothesize that tachycardia could be a manifestation of a high output state rather than a consequence of deconditioning. We audited records of adolescents presenting with long‐standing history of any mix of fatigue, dizziness, nausea, who underwent both head‐up tilt table test and maximal exercise testing with measurement of cardiac output at rest plus 2–3 levels of exercise, and determined the cardiac output (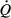) versus oxygen uptake (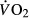) relationship. Subjects with chronic fatigue were diagnosed with POTS if their HR rose ≥40 beat·min^−1^ with head‐up tilt. Among 107 POTS patients the distribution of slopes for the 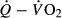, relationship was skewed toward higher slopes but showed two peaks with a split at ~7.0 L·min^−1^ per L·min^−1^, designated as normal (5.08 ± 1.17, *N* = 66) and hyperkinetic (8.99 ± 1.31, *N* = 41) subgroups. In contrast, cardiac output rose appropriately with 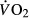 in 141 patients with chronic fatigue but without POTS, exhibiting a normal distribution and an average slope of 6.10 ± 2.09 L·min^−1^ 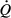 per L·min^−1^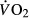. Mean arterial blood pressure and pulse pressure from rest to exercise rose similarly in both groups. We conclude that 40% of POTS adolescents demonstrate a hyperkinetic circulation during exercise. We attribute this to failure of normal regional vasoconstriction during exercise, such that patients must increase flow through an inappropriately vasodilated systemic circulation to maintain perfusion pressure.

## Abstract (precis)

e12122

Forty percent of postural orthostatic tachycardia syndrome (POTS) adolescents who, by definition have abnormal sympathetic control of HR and BP, demonstrate a hyperkinetic circulation during exercise. We attribute this to failure of normal regional vasoconstriction during exercise, such that patients must increase flow through an inappropriately vasodilated systemic circulation to maintain perfusion pressure.

## Introduction

Postural tachycardia syndrome (POTS) is defined by symptoms of orthostatic intolerance associated with a physical sign: an excessive increase in heart rate (HR) on orthostatic challenge (Medow and Stewart 2007). It was initially described in adults as a syndrome of orthostatic intolerance characterized by dizziness – sometimes syncope – fatigue, abdominal discomfort, and headache or other somatic pain. POTS is a heterogeneous disorder of unknown etiology with neuropathic, neurohumoral, or circulatory variants (Garland et al. 2007; Medow and Stewart 2007; Benarroch 2012; Raj 2013). Many patients with POTS limit physical activity whether due to pervasive fatigue and malaise or difficulty from orthostatic intolerance, presumably the reason why the majority of patients with POTS are deconditioned. Parsaik et al. (2012) found peak oxygen uptake (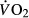) <85% predicted value in 90% of patients with POTS. Observations such as these prompt speculation that POTS is akin to a deconditioned state (Joyner and Masuki 2008; Lewis et al. 2013). Whereas these reports invariably studied adult patients and it is now recognized that diagnostic criteria for POTS in adolescents differ from adult (Johnson et al. 2010; Singer et al. 2012), deconditioning ought to have similar consequences or manifestations in adults or adolescents.

We previously reported excessive tachycardia during and after exercise in adolescents with POTS, compatible with deconditioning, but also noted that cardiac output rose more steeply than expected (Burkhardt et al. 2011). The heart functions as demand pump during exercise controlled principally by the tissues' need for oxygen and by the capacitance of the circulatory system. Blood flow through tissues is regulated by local metabolic requirement, and therefore must increase with exercise, leading to greater venous return to the heart. Distribution of blood flow is modulated as vessels dilate and constrict thereby increasing and decreasing resistance to flow depending on local need, orchestrated by the sympathetic nervous system (Charkoudian and Wallin 2014). Furlan et al. found that patients with chronic orthostatic intolerance exhibit discordant cardiac and vascular sympathetic control such that they had greater tachycardia but blunted rise in muscle sympathetic nerve activity accompanied by lesser vasoconstrictor response during head‐up tilt compared with controls (Furlan et al. 1998). It follows that disturbances in sympathetic nervous system control of circulation such as one sees in POTS could influence the HR and stroke volume (SV) responses to exercise.

Stewart and Montgomery (Stewart and Montgomery 2004) proposed a model of high‐, normal‐, and low‐flow POTS based on measurements of limb blood flow. If there were indeed high limb blood flow there must be high cardiac output, otherwise patients would present with swollen lower extremities. Some patients with POTS report vasoactive changes of cutaneous blood flow such as acrocyanosis or mottling rather than edema (Medow and Stewart 2007; Raj 2013). Our prior observations on cardiovascular responses to exercise in adolescents with POTS (Burkhardt et al. 2011) led us to postulate that some POTS patients may have a high‐flow state wherein 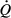 rises more steeply than expected with respect to 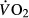, termed a hyperkinetic circulation (Haller et al. 1983). We hypothesized that there were at least two groups of adolescents with POTS – normal and high output or hyperkinetic – with unique cardiovascular responses to exercise such that a subgroup of adolescents whose head‐up tilt table test was positive would also show an amplified cardiac output response to exercise. We compare adolescents with POTS (nearly all of whom complained of fatigue) to chronically fatigued adolescents without POTS whose head‐up tilt table test was negative. Moreover, deconditioned patients – whether or not they had POTS – would show classical features of rapid HR and low SV during exercise. In contrast, patients with POTS who demonstrated a hyperkinetic circulation during exercise might achieve this high‐output state with relative tachycardia‐like normal‐flow POTS (consistent with abnormal head‐up tilt test) but with a normal SV. Therefore, a corollary hypothesis is that not all POTS patients with high exercise HR are *de facto* deconditioned.

## Methods

### Patient populations

**Figure 1.**
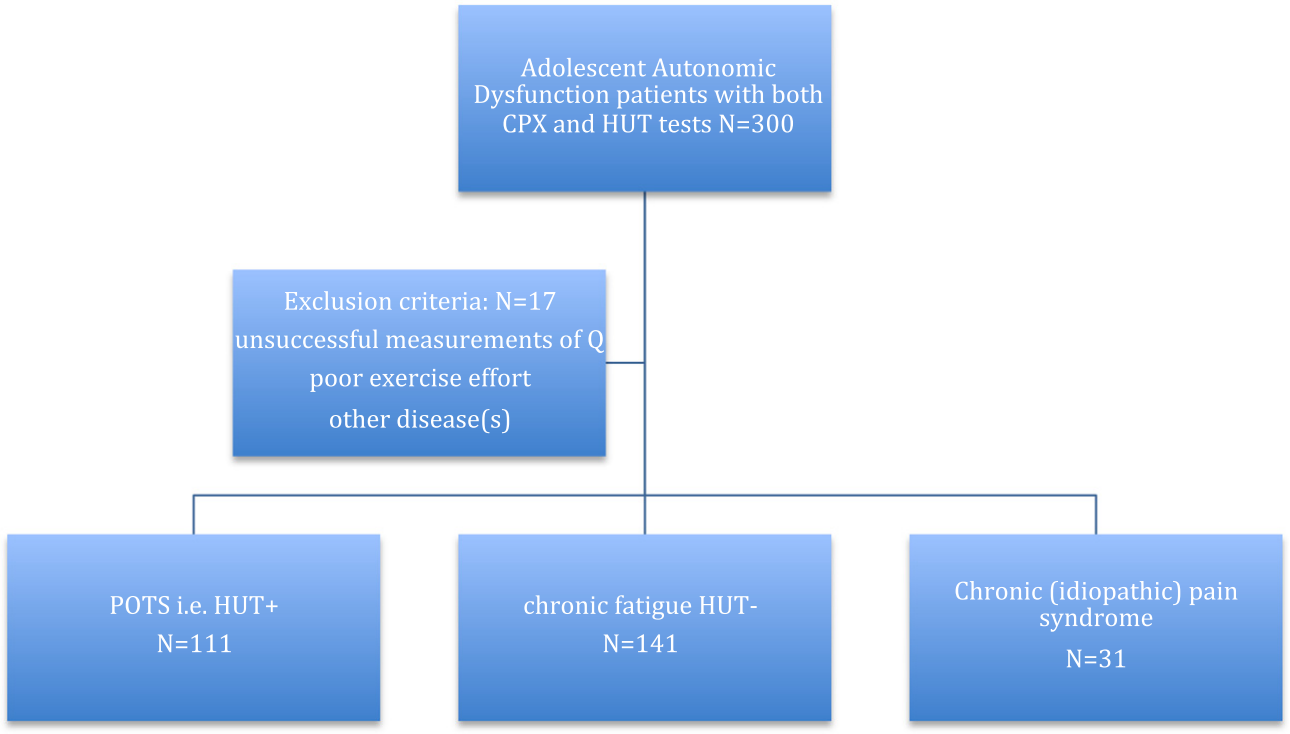
Flow diagram showing triage of patients with POTS, chronic fatigue, and exclusions.

We reviewed medical records of adolescents (12–19 years of age) seen in the Mayo Pediatric Diagnostic Referral Clinic from January 2010 to April 2012 with any mix of: chronic fatigue, dizziness, abdominal discomfort (nausea or pain), or other pain (headache, myalgia or arthralgia); who underwent both autonomic reflex and maximal exercise tests. We did not employ any particular set of inclusion criteria either when patients were triaged in clinic or in data abstraction, other than symptoms of *at least* 6 months duration. Thus, patients may or may not have fit the case definition (Fukuda et al. 1994) of chronic fatigue syndrome (CFS) but we did exclude those with alternative medical diagnoses explaining their symptoms. Patients were diagnosed with POTS if their ΔHR was ≥40 beats·min^−1^ during a 10‐min head‐up tilt table test performed according to standard‐of‐care methods at Mayo Clinic (Thieben et al. 2007). Patients whose ΔHR fell below that threshold *and* whose chief complaint or reason for seeking medical evaluation was chronic fatigue were assigned to the chronic fatigue group to serve as a comparison group specifically to address issues and differences in exercise cardiac output measurement and results. We anticipated this group would maintain a normal 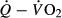 response since deconditioning has not been shown to alter this function, and because autonomic regulation of the cardiovascular system ought to be intact. The breakdown of patients triaged, inclusions, and exclusions, are shown in Figure 1. Testing was conducted for clinical indications and informed consent was, therefore, not required. Mayo Clinic Institutional Review Board approved the study.

### Measurements

Subjects performed a maximal cardiopulmonary exercise test on a cycle ergometer according to the Godfrey protocol of 1‐min incremental workloads (Godfrey 1974). Ventilation and gas exchange was measured breath‐by‐breath at rest and throughout exercise using MedGraphics CPX/D (Breeze software; MGC Diagnostics, St. Paul, MN) which employs a Pitot tube to measure flow, electronically integrated to give minute volume (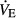), yielding values for 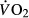, carbon dioxide output (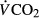) computed as means over the final 15–20 sec of each workload. Cardiac output was measured using a closed circuit, acetylene–helium rebreathe technique (Triebwasser et al. 1977). Exhaled gases were measured by mass spectrometry (Perkin‐Elmer, Pomona, CA). 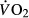 and 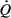 were measured at rest with the subject seated on the cycle ergometer; 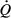 was also measured during the first workload then at alternate workloads at the discretion of the supervising physician in order to obtain at least 2–3 measures of 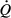 during exercise without causing the patient undue distress from rebreathing. Experience from previous testing had taught us that some patients from this population became visibly upset or simply came off the mouthpiece if rebreathing was done during heavy exercise. Heart rate was monitored with a 12‐lead continuous ECG (Quinton Cardiology Systems, Inc., Bothell, WA). Blood pressure was measured by auscultation prior to each 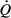 determination plus every other subsequent work increment. Oxygen saturation was continuously monitored by pulse oximetry (OxiMax N‐600X Nellcor, Hayward, CA). Patients were strongly encouraged to exercise to voluntary exhaustion but given their chronic fatigue state, a minority of patients failed to achieve the usual, accepted criteria (e.g., respiratory quotient >1.1 or HR >180 beats·min^−1^) for a maximal test. Their data were excluded from peak 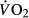 analysis but included in cardiac output analysis. Peak 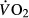 was determined as the highest achieved 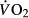 sustained over 20 sec and peak oxygen pulse was calculated as 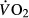/HR at peak exercise. Peak 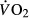 results were compared with normal, age‐appropriate, values (NHANES) from a US adolescent population (Eisenmann et al. 2011). Deconditioning was defined as peak 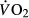 less than sex‐specific 10‰. Stroke volume was computed as 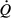 divided by HR and normalized for body surface area to give stroke volume index (SVI) in mL·m^−2^). The preponderance of females precluded separate analyses by sex. Some patients with POTS hyperventilate (Stewart et al. 2006) which may contribute to dizziness. Therefore, ventilatory response was examined and characterized by intercept and slope: end‐tidal PCO_2_ at the ventilatory anaerobic threshold (Beaver et al. 1986); and change in ventilation versus change in CO_2_ output during subthreshold exercise (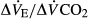), respectively.

### Statistical methods

Values are reported as mean ± SD, or median (IQR) as appropriate. Linear regression was used to compute 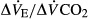 and 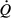 versus 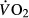. Correlations were assessed using the Pearson correlation coefficient (*r*) or Spearman correlation coefficient (*r*_S_) for nonnormal data (e.g., ∆HR during head‐up tilt. Comparisons between group means were tested using two‐sample *t‐*test, whereas proportions were compared with *χ*^2^; *P*‐values of <0.05 were considered significant.

## Results

### POTS patients

Patient anthropometric data are shown in Table 1. None were anemic. Presenting complaints included dizziness (84%), fatigue (71%), headache (74%), nausea (46%), abdominal pain (45%), and syncope (26%). Median (IQR) duration of symptoms was 22 (DeLorenzo et al. 1998; Stewart and Montgomery 2004) months. All 111 patients with POTS, by definition, exhibited ≥40 beats·min^−1^ rise in HR during head‐up tilt. Median (IQR) ∆HR was 46 (Trevisani et al. 1999; Rowland and Whatley Blum 2000) beats·min^−1^; ∆BPsys with head‐up tilt was −12 (−6, −18) mmHg, but 18% experienced a drop ≥20 mmHg. Two‐thirds of patients were symptomatic during head‐up tilt, causing premature test termination in 10%, but only one had syncope.

**Table 1.** Selected anthropometric, and resting circulatory data, split according to diagnosis, sex, and slope of the cardiac output – oxygen uptake relationship during exercise (POTS only)

	POTS				Chronic fatigue	
	Normal flow		Hyperkinetic			
	M	F	M	F	M	F
*N*	16	50	7	34	38	103
Age (years)	15 ± 3	16 ± 2	16 ± 1	15 ± 2	14.5 ± 1.7	15.8 ± 1.5
Height (cm)	174 ± 14	167 ± 8	183 ± 12	165 ± 7	171 ± 11	163 ± 7
Weight (kg)	63.2 ± 13.5	60.1 ± 12.5	76.5 ± 16.8	58.4 ± 11.0	70.2 ± 18.7	61.3 ± 13.0
BSA (m^2^)	1.76 ± 0.25	1.67 ± 0.18	1.98 ± 0.25	1.64 ± 0.17	23.7 ± 4.7	1.65 ± 0.17
BMI (kg·m^−2^)	20.7 ± 2.7	21.5 ± 3.7	22.8 ± 4.3	21.2 ± 3.4	1.81 ± 0.27	23.1 ± 4.1
HR (beat·min^−1^)	96 ± 17	90 ± 17	91 ± 11	99 ± 18	90 ± 14	94 ± 18
CI (L·min^−1^·m^−2^)	3.75 ± 0.89	3.65 ± 1.15	3.93 ± 0.85	3.34 ± 0.84	3.67 ± 0.76	3.77 ± 1.14
Stroke volume index (mL·m^−2^)	41 ± 12	37 ± 11	41 ± 9	36 ± 10	40 ± 11	39 ± 11
Hgb (g·dL^−1^)	14.6 ± 1.1	13.2 ± 0.6	14.9 ± 0.8	13.0 ± 0.7	14.5 ± 1.1	13.0 ± 0.9

Maximal exercise data are shown in Table 2. There was no correlation between peak 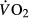 (mL·kg^−1^·min^−1^) and ∆HR during head‐up tilt. Peak 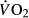 was <10‰ in 56% of females and in 65% of males (*χ*^2^ = 0.6791, *P = *0.41). The hyperkinetic POTS group had lower peak 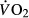 (Table 2) and there was weak (*P = *0.05) correlation between peak 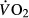 (mL·kg^−1^·min^−1^) and 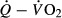 slope. The principal reason for termination of the exercise test was (*N*): generalized fatigue (Rowland and Whatley Blum 2000), sore legs (Higginbotham et al. 1986), dizziness (Fukuda et al. 1994), dyspnea (di Prampero 2003), none specified (Charkoudian and Wallin 2014). End‐tidal PCO_2_ at the ventilatory anaerobic threshold was the lowest in the patients who ceased exercise complaining of dizziness, averaging 37.9, 38.0, 34.4, 37.4, 37.5 mmHg, respectively (*P *=**0.01). The slope 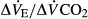 during subthreshold exercise averaged 30.7 ± 4.5 and correlated inversely end‐tidal PCO_2_ at the ventilatory anaerobic threshold (*r *= −0.63, *P *<**0.0001). Furthermore, 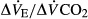 correlated with (*P < *0.0001), and explained 17% of the variance of, peak 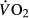.

**Table 2.** Selected peak exercise data in patients, split according to diagnosis, sex, and slope of the cardiac output–oxygen uptake relationship during exercise (in POTS patients only)

	POTS				Chronic fatigue	
	Normal 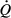		Hyperkinetic			
	M	F	M	F	M	F
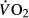						
mL·kg^−1^·min^−1^	33.8 ± 8.2	31.3 ± 6.2*	34.0 ± 7.6	27.9 ± 6.5*	36.1 ± 8.2	27.4 ± 6.0
L·min^−1^	2.11 ± 0.60	1.86 ± 0.44	2.51 ± 0.38	1.63 ± 0.41	2.46 ± 0.57	1.64 ± 0.32
Work						
Watts	150 ± 50	138 ± 30	184 ± 315	125 ± 31	170 ± 46	127 ± 26
% predicted	60 ± 23	77 ± 17	59 ± 14	73 ± 23	71 ± 19%	76 ± 25%
HR						
Beats·min^−1^	186 ± 17	187 ± 11	190 ± 11	189 ± 8	189 ± 10	187 ± 13
O_2_ pulse						
mL·beat^−1^	11.3 ± 3.0	9.3 ± 2.3	11.7 ± 3.8	8.7 ± 2.2	13.1 ± 3.2	8.8 ± 1.6
VAT						
% peak 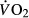	58 ± 9	58 ± 8	53 ± 6	58 ± 7	55 ± 12%	61 ± 8%

* *P < *0.02 girls with hyperkinetic circulation versus normal cardiac output.

**Figure 2.**
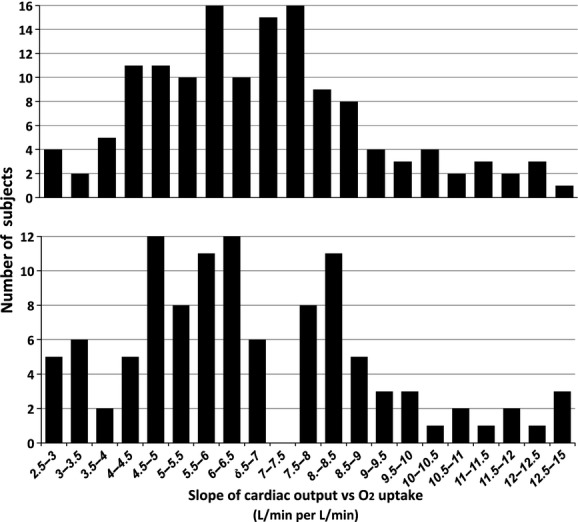
Frequency distribution of slopes of cardiac output versus oxygen uptake from rest to exercise in patients with POTS (lower panel) or chronic fatigue (upper panel).

Data to determine slope of the 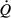 versus 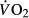 relationship during light to moderate exercise were obtained in 107 patients: rest plus one exercise 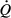 in 17% of subjects (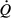 in one patient measured successfully only at 25 and 75 watts); rest plus two measurements of exercise 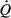 in 65%; and rest plus three or four measurements of exercise 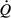 in the remaining 18%. Mean *r* value of linear regression analyses was 0.90. Figure 2 shows the frequency distribution for 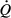 versus 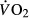 slopes for all subjects. The distribution obtained from the POTS patients (lower panel) was skewed with a prominent tail reaching supranormal values. Skewness was verified statistically because the 95% confidence interval for a difference between mean (6.69) and median (6.07) values should fall within ± 0.4 for a sample of *N* = 100 (Doane and Seward 2011). There appeared to be two clusters in the distribution from our POTS population, with a split at ~7.0. On average, *Q* rose by 5.06 ± 1.17 L·min^−1^ versus 9.61 ± 1.31 L·min^−1^ for each L·min^−1^ rise in 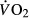 for each cluster‐mode, which we label normal (*N* = 66) and hyperkinetic (*N* = 41) POTS subgroups. We were unable to find a correlation between individual slope for 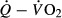 and ∆HR during head‐up tilt table testing.

**Figure 3.**
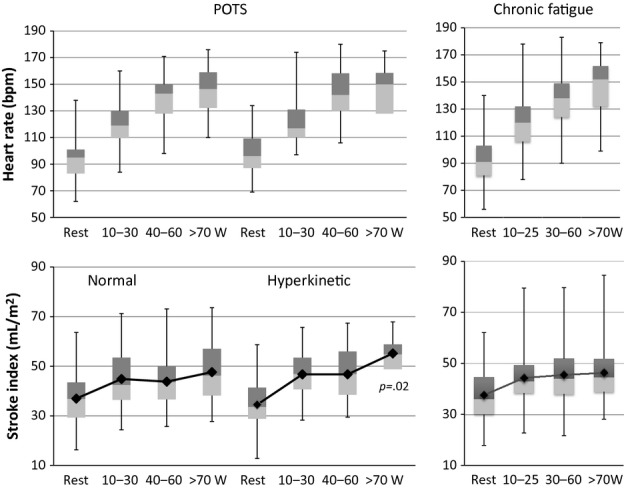
Box and whisker plots of HR (above) and stroke volume index (below) at rest, and during progressive exercise shown separately for normal and hyperkinetic cardiac output groups of POTS patients; and for patients with chronic fatigue.

**Figure 4.**
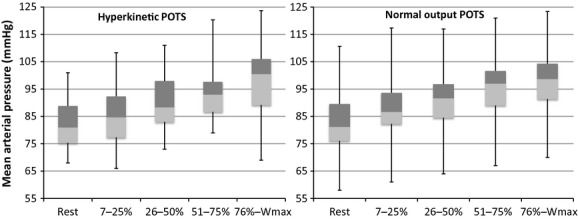
Box and whisker plots of mean arterial blood pressure at rest and during progressive exercise (abscissa is percent maximum work capacity) shown separately for normal cardiac output and hyperkinetic groups of POTS patients.

Mean resting SVI for all POTS patients was 36 ± 11 mL·m^−2^ and rose significantly in light exercise to 46 ± 11 mL·m^−2^ before leveling off in moderate exercise at 47.5 ± 11.5 mL·m^−2^. Both normal output and hyperkinetic groups showed the expected rise in SVI with light exercise and moderate exercise but mean SVI was lower (*P *=**0.02) during heavy (~⅔ peak 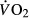) exercise in the normal flow (47 ± 11 mL·m^−2^) versus the hyperkinetic (56 ± 7 mL·m^−2^) POTS subgroup (Fig. 3). Exercise HR trajectories were similar (Fig. 3). Mean blood and pulse pressures also followed similar trajectories during exercise in normal output and hyperkinetic POTS patients (Fig. 4).

### Chronic fatigue patients

Patient anthropometric data are shown in Table 1. There were 141 patients in the chronic fatigue group, all of whom exhibited <40 min^−1^ rise in HR during HUT: median (IQR) ∆HR was 28 (Haller et al. 1983; Lewis et al. 2013) beats·min^−1^. Reported symptoms included fatigue (100%), dizziness or “brain fog” (85%), headache (70%), nausea or abdominal pain (48%), and other pain for example, chest, muscles, joints (50%); and median (IQR) symptom duration was 29.5 (Stewart and Montgomery 2004; Fu et al. 2010) months. Maximal exercise data are shown in Table 2. Only 39% of males had peak 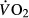 >10‰ (~36.8 mL·kg^−1^·min^−1^) compared to 32% of females who exceeded 30.8 mL·kg^−1^·min^−1^ threshold (*χ*^2^ = 0.31, *P = *NS).

Slope of the 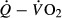 relationship from rest to moderate exercise was determined in 137 patients: two measurements of 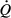 in 23%; three in 55% of subjects; four measurements of 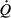 in the remaining 22%. The frequency distribution of 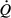 versus 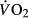 (Fig. 2, upper panel) was normal with a mean slope of 6.10 ± 2.09 L·min^−1^ 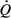 per L·min^−1^ rise of 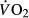, significantly less than the mean slope for POTS patients (*P *=**0.039). Mean *r* of individual linear regression analyses was 0.92. Exercise HR trajectory is shown in (Fig. 3) and was very similar to that in POTS. SVI in chronic fatigue patients rose with increasing work (Fig. 3) but reached a plateau no different than normal output POTS patients, exceeding 50 mL·m^−2^ in only 46 (33%) subjects.

## Discussion

This study on the cardiac output response to exercise in adolescents with POTS supports the hypothesis that there are at least two distinct populations based on blood flow characteristics: one that maintains the normal coupling between 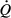 and 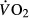 plus another in whom 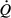 rises more than expected with respect to 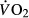. This hyperkinetic state was achieved by relative tachycardia but with either low or *normal* SV response to exercise. Deconditioning is typically inferred from an incremental exercise test to voluntary exhaustion when a subject fails to achieve a reference peak 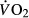, has a low ventilatory anaerobic threshold, plus an exaggerated HR response such that O_2_ pulse is low (Ross 2003). The underlying presumption is that 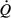 rises normally with 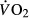, and therefore, if HR is high then SV must be low. Our results show this reasoning does not apply to at least one‐third of adolescents with POTS. We also found that patients who hyperventilated were more likely to cite dizziness as a limiting exercise, which contributed to lower peak 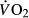.

In their review of circulatory control during exercise, Rowell and O'Leary (1990) posed the fundamental question of whether the primary error corrected by the (normally functioning) sympathetic nervous system during exercise is a mismatch between blood flow and metabolism – a flow error – or a mismatch between vascular conductance and cardiac output – a pressure error. Inasmuch as blood pressure was very similar between normal flow and hyperkinetic POTS, our results imply that blood pressure is indeed *the* regulated variable during exercise, even at the expense of cardiac output. We demonstrated a linear 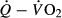 relationship but the slope of that function was abnormally high in 40% of our patients. 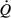 is related to both the degree of muscle activation and to 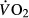 during leg exercise (Dufour et al. 2007), rising at an average rate of ~5–6 L·min^−1^ 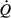
per L·min^−1^ rise in 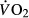 at least during submaximal exercise in adults (Astrand and Rodahl 1970) and children (Godfrey 1974). Slopes for the 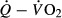 relationship in healthy adults vary in a normal distribution from 5.5 to 10.3 (Yamaguchi et al. 1986). Intersubject variability in the slope of the 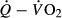 relationship is due to unique variations in resting or exercise hemodynamics and to arteriovenous O_2_ content differences, particularly during exercise. The circulatory response to exercise in our “control” adolescents with chronic fatigue was similar to that reported in normal individuals, and different from POTS adolescents by virtue of its normal distribution with a normal mean value. In contrast, patients with POTS comprised a skewed distribution with statistically significant differences between mean and median, and with a higher group mean value than a large sample of chronically fatigued adolescents with negative head‐up tilt table test. Hyperkinetic circulation has been described in a few other disorders (Lonsdorfer et al. 1983; Trevisani et al. 1999) but none related to POTS. High 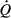 for 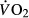 implies reduced O_2_ extraction at the level of exercising muscle, such as one might see if a deficiency in metabolic capacity existed (Haller et al. 1983). Indeed, Holmgren et al. (1957) showed precisely this (see Fig. 5) in adults with “vasoregulatory asthenia,” likely patients who would now be diagnosed as POTS. Exercise may not be limited by O_2_ delivery but rather by its utilization in working muscle in such patients. This impairment of peripheral O_2_ transfer and uptake explains decrements in supine peak 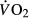 and that portion of upright peak 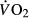 not explained by classical deconditioning (Bringard et al. 2010).

Bicycle exercise performed in this study utilizes large muscle groups, and more than 70% of 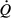 may go to contracting muscles during maximal exertion (Calbet et al. 2007). In order to achieve this, blood must be diverted from nonessential vascular beds to preserve flow to essential tissues and organs, failure of which would deprive the working muscles of vital blood supply when needed most. The diaphragm requires relatively rich blood supply at high levels of ventilation. Hyperventilation may thus deprive working leg muscles of blood flow and limit performance (Harms et al. 1997). Moreover, changes in local perfusion produce the same effects proportionately on muscle contractility such that muscle fatigue would occur more rapidly without an adequate (local) exercise pressor response (Luu and Fitzpatrick 2013). Hyperkinetic circulation could in essence become a “steal” phenomenon that, coupled with hyperventilation, explained almost 20% of the variance in low peak 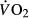 in a multivariate regression model. Stroke volume is the principal determinant of maximum cardiac output, in turn a major determinant of peak 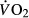 (Bassett and Howley 2000). If one subscribes to the paradigm that maximum 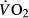 is ultimately limited by O_2_ transport (di Prampero 2003), it follows that one reason for low peak 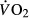 in some POTS patients is *not* that they are unable to muster sufficient 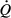 but rather they are unable to direct flow to where it is most needed. Affected patients comprise a heterogeneous population who share certain abnormalities of blood flow regulation (Garland et al. 2007; Medow and Stewart 2007; Benarroch 2012; Raj 2013). Stewart (Stewart and Montgomery 2004) described a model of POTS with “low‐flow,” “normal‐flow,” and “high‐flow” state. Hyperkinetic circulation could be a manifestation of their high‐flow group, and it is conceivable that the low‐flow patients were buried in subjects whose 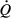 rose slightly less than expected for 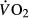 (left tail of Fig. 2 lower panel). Our novel observation elaborates current models of POTS and injects a cautionary note on invocation of deconditioning in this population.

Relative tachycardia and low SV during exercise are considered hallmarks of cardiovascular deconditioning, yet many of our POTS patients achieved normal exercise SV. There are few normal reference values for exercise SVI in adolescents (Rowland and Whatley Blum 2000; Pianosi 2004) but peak values approximate 50 mL·m^−2^, close to values found in adults (Higginbotham et al. 1986). Conventional wisdom is that SV rises at onset of exercise, reaches a plateau in moderate exercise, but may fall during heavy exercise in adults (Gonzalez‐Alonso 2008; Warburton and Gledhill 2008). There is a dearth of such data in pediatric subjects though SV may continue to increase right up to peak exercise in healthy children and adolescents (Pianosi 2004). Rowland et al. found no differences in inotropic or lusitropic responses to progressive exercise despite higher SVI in adolescent athletes versus nonathletes, and concluded that greater aerobic fitness in trained subjects reflected volume expansion of the circulation rather than enhanced ventricular function (Rowland et al. 2009). Volume depletion is a cardinal feature of the deconditioned state (Coyle et al. 1986) and POTS (Benarroch 2012; Raj 2013) and may account for much of the observed reduction in heart size in patients with CFS (Miwa and Fujita 2008; Hurwitz et al. 2010). We reported slower HR recovery post‐exercise – ostensibly a marker of deconditioning – in patients with POTS (Burkhardt et al. 2011), but HR recovery immediately after exercise is a function of a decline of sympathetic drive coupled with reactivation of parasympathetic drive (Imai et al. 1994). Though we did not measure intravascular volume in our patients, one could propose, an alternative, cogent, and coherent, interpretation of these observations. Development of the POTS phenotype begins with abnormal sympathetic regulation of volume status via renin‐angiotensin system (Garland et al. 2007) leading to volume depletion. At this point, little or no effect on inotropy results and SV is preserved but altered sympathetic modulation of the metaboreflex results in hyperkinetic circulation with normal SV in those with positive head‐up tilt table test. Low peak 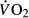 can supervene at any time, depending on premorbid peak 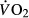 but will be exacerbated should a given individual develop a hyperkinetic circulation. Once the deconditioning spiral has begun, fatigue and dizziness perpetuate it culminating in the small heart associated with POTS (Fu et al. 2010; Parsaik et al. 2012).

There is much overlap between morbidity due to POTS and CFS (Freeman and Komaroff 1997; Schondorf et al. 1999), and some consider POTS to be a subset of CFS (Lewis et al. 2013). Cardinal symptoms of CFS are increased malaise or extreme exhaustion following physical exertion or mental activity, hypersomnolence, difficulties with memory and concentration, persistent muscle pain, joint pain, and headache, for which no cause can be found (http://www.cdc.gov/cfs/symptoms/). The overall prevalence of CFS was estimated at 235/100,000 persons (Reyes 2003), higher among women (373/100,000) than among men (83/100,000). The prevalence of CFS among adolescents is lower (Jones et al. 2004) though it does occur in the pediatric population (http://www.cdc.gov/cfs/symptoms/ accessed June 21, 2014). POTS typically develops between 15 and 25 years of age with a striking female predilection (Johnson et al. 2010; Benarroch 2012). There are >500,000 patients in the United States with POTS (Garland et al. 2007), with one estimate of the prevalence of POTS is 170/100,000 based on the investigators' finding that ~40% of patients with CFS have POTS. The degree of deconditioning was similar in patients with POTS as in patients with chronic fatigue (Table 2), yet this amplified circulatory response to exercise was seen almost exclusively in patients who had a ΔHR >40 beats·min^−1^ with head‐up tilt. This test threshold differentiated those with POTS and distinguished them from chronically fatigued adolescents with simple deconditioning whose regulation of circulation remained intact. Left ventricular mass is lower and pulse higher in CFS patients than in healthy controls, changes consistent with physical deconditioning and/or altered sympathetic‐parasympathetic balance (DeLorenzo et al. 1998; Miwa and Fujita 2008). Yet, the circulatory response to exercise should be no different in patients with POTS versus those with chronic fatigue *if* POTS were merely a consequence or signature of deconditioning as some have argued (Fu et al. 2010; Parsaik et al. 2012). In contradistinction, we submit that whereas *most* patients with POTS suffer from chronic fatigue and have a similar circulatory response to exercise – that of cardiovascular deconditioning – many do not fit this bill and are not merely one end of a chronic fatigue spectrum.

Limitations of our study include the fact that 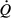 measurements were restricted to light or moderate exercise. There is some evidence that the 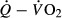 relationship is curvilinear, leveling off in heavy exercise in more fit individuals, implying additional O_2_ consumption is achieved by greater O_2_ extraction, as suggested by Beck et al. (2006). Their subjects in the nonlinear group had higher SV and lower HR at rest, consistent with data they obtained in more fit individuals. While we may have overestimated the change in 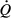 for 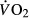 by limiting observations to subthreshold exercise, even Beck et al. concur that in all their subjects, the mean slope for 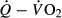 was 5.2 during light to moderate exercise. Different fitness levels do not explain the divergent slopes for the 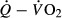 relationship for the following reasons: (1) patients in each cluster exhibited different trajectories for 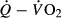 during exercise at low‐moderate intensities; and (2) differences in HR and SV were not apparent at rest in our groups of patients, unlike Beck et al. The normal relationship between 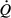 and 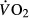 during exercise is well established (Astrand and Rodahl 1970), though it may become curvilinear and asymptotic near maximal exercise (Beck et al. 2006). Rebreathing methods become intolerable for subjects breathing at high levels of exercise, which limit observations to subthreshold exercise. Designation of deconditioning relied on a large national reference (NHANES) sample but was justified as it truly reflected the make‐up of our study population. We did not have a true, “healthy” control population but instead used patients with chronic fatigue. We submit this is an appropriate control population in that they exhibited nearly identical symptoms and differed only inasmuch as their head‐up tilt table test was normal/negative.

In conclusion, most adolescents with POTS or chronic fatigue are deconditioned, and show the expected cardiovascular manifestations of low SV coupled with high HR. Measurement of cardiac output during exercise provided additional data regarding hemodynamic changes by differentiating CFS from some POTS patients and allowed better characterization of their pathophysiology and fitness level. This should lead to phenotyping this heterogeneous population and may assist in tailoring therapy, for example, *β*‐blockade versus *α*‐agonist, and offering appropriate suggestions for exercise prescription. Physical inactivity leading to deconditioning appears to be a final common pathway for many disorders. It follows that exercise training ought to be an effective remedy for, and become incorporated into the management of, POTS and CFS. That said, an intriguing possibility is that simply exercising in the supine posture might restore a normal 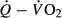 relationship in patients with hyperkinetic POTS.

## Acknowledgments

Authors are grateful to A. L. Weaver MSc, for providing statistical consultation services.

## Conflict of Interest

None declared.
